# Reporting Antimicrobial-Related Adverse Drug Events in Jordan: An Analysis from the VigiBase Database

**DOI:** 10.3390/antibiotics12030624

**Published:** 2023-03-21

**Authors:** Nizar Mahmoud Mhaidat, Sayer Al-Azzam, Hayaa Abdallah Banat, Jaber Mohammad Jaber, Mohammad Araydah, Osama Y. Alshogran, Mamoon A. Aldeyab

**Affiliations:** 1 Clinical Pharmacy Department, Faculty of Pharmacy, Jordan University of Science and Technology, Irbid 22110, Jordan; 2 Jordan Food and Drug Administration (JFDA), Amman 11181, Jordan; 3 Princess Basma Teaching Hospital, Irbid 22110, Jordan; 4 Department of Pharmacy, School of Applied Sciences, University of Huddersfield, Huddersfield HD1 3DH, UK

**Keywords:** VigiBase, antimicrobial stewardship, antimicrobial resistance, adverse events, adverse reactions, pharmacovigilance

## Abstract

This study aims to assess the reporting of antimicrobial-related adverse drug events (ADEs) in Jordan between 2003 and 2022. Data regarding the antimicrobial-related ADEs were extracted from the WHO’s global database (VigiBase) by the Rational Drug Use and Pharmacovigilance Department at the Jordan Food and Drug Administration (JFDA). A total of 279 Individual Case Safety Reports (ICSRs) were recorded. The number of ICSRs increased from 2019 onwards (219 out of 279 cases). This increase in the reported ADEs was influenced by the actions of the JFDA, including the introduction of electronic reporting forms, updating the national pharmacovigilance guidelines, which encouraged adverse drug reactions reporting, the implementation of the AMR-national action plan, the encouragement to report due to COVID-19 vaccine, and the continuous awareness campaigns and training programs. Skin and subcutaneous tissue disorders (*n* = 105; 19.48%) were the most reported antimicrobial-related ADEs. The highest number of ADEs was reported for tetracyclines (*n* = 101; 18.74%) followed by fluoroquinolones (*n* = 54; 10.02%), third-generation cephalosporines (*n* = 48; 8.9%), and carbapenems (*n* = 42; 7.79%). From the top 10 consumed antibiotics, the number of ADEs in patients who consumed Watch group antibiotics (97 ADEs) was higher than those who consumed Access group antibiotics (28 ADEs). The findings highlight the need to monitor and rationalize the use of Watch antibiotics. Enhanced reporting of antimicrobial-related adverse drug reactions is needed to inform antimicrobial stewardship and improve the pharmacovigilance system in Jordan.

## 1. Introduction

For every member of society, ensuring the safety of pharmaceuticals is a crucial goal that must be attained, although, in many developing nations, this goal is severely disregarded [1,2]. Adverse drug events (ADEs) are considered the most frequent kind of adverse event that patients encounter [3]. An adverse drug event (ADE) is defined as “any injury occurring during the patient’s drug therapy and resulting either from appropriate care or from unsuitable or suboptimal care” [4]. Based on this definition, ADEs comprise adverse drug reactions (ADRs) resulting from taking medications as recommended and any damage resulting from inappropriate medication use, or medication errors [5]. Despite the enormous benefits of various pharmaceuticals in managing serious illnesses, ADRs are still considered one of the major causes of harm to people’s lives [6,7]. As one of the most common global causes of injury, where the primary outcome is the death of thousands of patients each year, ADEs are one of the main issues concerning patient safety [8]. ADRs have an enormously negative impact on healthcare systems by interfering with patient care [9]. These exceedingly harmful effects include increased morbidity and mortality rates, an increased need for admission, thus, increasing the financial burden, and death [9].

“The science and activities relating to the detection, assessment, understanding, and prevention of adverse effects or any other medicine/vaccine-related problem” fall under the term “pharmacovigilance” [10,11]. Enhancing public health and safety, regarding the use of various medications, is the main goal of pharmacovigilance [12]. Furthermore, pharmacovigilance aims to evaluate the benefits and hazards of adverse drug events, and reactions to medication usage, to promote the safe, rational, and more efficient use of medications [12].

According to research by the Institute of Medicine, ADRs cause at least 7000 fatalities annually in the United States (US), with expenditures ranging from 17 to 29 billion USD annually [13]. In addition, in the United Kingdom, approximately 6.5% of hospitalized patients experienced ADRs, with an approximate annual cost of 466 million GBP [14]. In a systematic review by Silva and colleague, it was found that the ADE-related hospitalization rate ranged from 9.7 to 383 per 100,000 population and the mortality rate ranged from 0.1 to 7.88 per 100,000 population [15]. Additionally, a meta-analysis of descriptive cohort studies found that ADRs cause 4.9 to 41.3% of annual hospital admissions worldwide [2,10]. In a prospective cohort study conducted over 4 years, ADEs compromised 87% of the visits to the emergency department (ED) and led to hospitalization and death in 49.3 and 2.2% of the population, respectively [16]. In a study conducted in Jordan, ADEs were encountered in 10.8% of hospital admissions and more than half (55.3%) of these adverse reactions were categorized as “unpreventable” [17]. The fact that roughly half of the ADE-related hospital readmissions were found to be avoidable emphasizes the significance of identifying and disclosing medication-related events [18,19,20]. Through signal detection and regulatory actions, early recognition of ADEs can limit serious clinical outcomes, lessen the related economic burden, and increase public safety. Furthermore, pharmacovigilance hospital systems can be used to process ADE-related information, which can be used to alter, renovate, and evolve treatment guidelines by detecting gaps in the medication usage knowledge and creating new risk management strategies [21].

It was observed that antimicrobial-related ADRs constitute a major part of drug-related adverse reactions in multiple previous studies [3,22,23]. In a recent study involving 1488 hospitalized adult patients, who received systemic (oral or intravenous) antibiotic treatment, it was found that 20% encountered at least one antibiotic-related ADE [24]. In addition, ADEs were associated with 20% of the non-clinically justified antibiotic regimens [24]. According to one study, conducted between 2011 and 2015 in the United States (US), 145,490 emergency department (ED) visits among US people aged 20 years or older were linked to antibiotic-associated ADEs [25]. Since antimicrobials may induce a variety of ADEs and there is growing concern regarding antimicrobial resistance (AMR), antimicrobial monitoring has taken on special importance [24,26]. Moreover, newly developed antimicrobials are hitting the market and, thus, need to be closely monitored [27]. If an ADE is reported after antimicrobial consumption, then, pharmacovigilance becomes a useful tool, which could help deal with issues associated with the use of antimicrobials, such as AMR [28]. The primary goals of antimicrobial stewardship programs include the optimization of proper antibiotic use, to enhance clinical outcomes, and the reduction of unintended side effects, such as the development of resistance and toxicity [29]. These goals can be implemented in clinical practices to prevent and reduce antibiotic-related ADEs, thus, promoting patient safety [30].

The World Health Organization (WHO) constructed a global program to monitor various drugs and facilitate the reporting of ADRs, known as the Program for International Drug Monitoring (WHO PIDM) [31,32]. Program members submit reports of suspected ADRs to the WHO’s database, VigiBase, which is a global database responsible for analyzing reports of suspected harm caused by medicines. The term “Individual Case Safety Report” (ICSR) refers to a document with a particular format for the reporting of one or more suspected adverse reactions to a medication, which happen in a single patient at a particular period [33]. The WHO PIDM is the world’s single-largest database on drug safety and is supported and maintained by the Uppsala Monitoring Centre, an international drug monitoring center situated in Sweden [34].

This study aims to assess the reporting of antimicrobial-related ADEs in Jordan between 2003 and 2022. The main objectives of this study were to describe: (a) the trends in the total number of ICSRs during the study period, (b) the seriousness and measures taken for antimicrobial-related ADEs, (c) the types of antimicrobial-related ADEs, (d) the antimicrobial groups associated with the most reported ADEs, (e) the ADEs reported from the top ten consumed antibiotics in Jordan, and (f) the relationship between the AWaRe categorization of antibiotics and the reporting of ADEs.

## 2. Results

### 2.1. Trends in the Number of ICSRs

**Figure 1:**
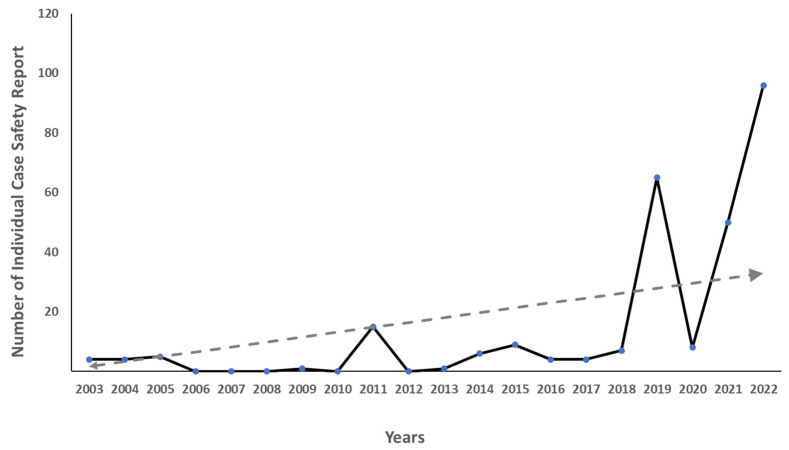
Trends in the number of Individual Case Safety Reports (ICSRs) in Jordan between 2003 and 2022.

As demonstrated in Figure 1, the number of ICSRs was at a steady level (4–5 cases) between 2003 and 2005, then, the number of ICSRs dropped to zero, and remained the same between 2006 and 2010, with exception of 2009, where once case was recorded. The number of ICSRs experienced a sudden increase, reaching 15 cases in 2011 before returning to 0 cases in 2012. A rise in the number of ICSRs was observed between 2013 and 2015 (from 1 to 9 cases), followed by a small drop in 2016 and 2017 (4 cases for each year), before a minor increase in 2018 (7 cases). After 2018, significant elevations and drops in the ICSR count were observed. The years 2019 and 2022 recorded the highest numbers of ICSRs (65 and 96 cases, respectively) in the whole study period.

### 2.2. Demographics and General Characteristics

A total of 279 ICSRs, regarding antimicrobial-related ADEs, were extracted from the VigiBase database. Patients were categorized into three age groups (Table 1). The number of patients aged between 18 and 64 years old (*n* = 121; 43.37%) was higher than patients aged under 18 years old (*n* = 68; 24.37%), and those aged 65 years old or older (*n* = 24; 8.6%). Furthermore, the number of females (*n* = 136; 48.75%) was higher than males (*n* = 100; 35.84%). Most of the antimicrobial-related ADEs were reported by physicians (*n* = 103; 36.92%) and pharmacists (*n* = 84; 30.1%).

### 2.3. Chemical Subgroup of Drugs Included in the Search

A total of 60 antimicrobial products were included with 539 ADEs (Table 2). The chemical subgroups of antimicrobials with the highest number of products, included in this cohort, were antifungals (*n* = 12), followed by antivirals (*n* = 8). The highest number of ADRs was recorded for tetracyclines (*n* = 101), followed by fluoroquinolones (*n* = 54), third-generation cephalosporines (*n* = 48), and carbapenems (*n* = 42). The lowest count for antimicrobial-related ADEs was recorded in antiprotozoals (*n* = 1) and other antibacterial (*n* = 1).

### 2.4. Seriousness and Actions Taken for Antimicrobial-Related ADEs

Of the study population, 120 (43.01%) patients were categorized as experiencing serious antimicrobial-related ADEs (Table 3). Most patients involved in this cohort experienced antimicrobial-related ADEs that either caused or prolonged hospitalization (*n* = 58; 20.79%). Furthermore, life-threatening antimicrobial-related ADEs were experienced by 10 (3.58%) patients, and death was encountered by 13 (4.66%) patients. Other criteria for seriousness were as follows: disabling/incapacitating (*n* = 1; 0.36%), congenital anomaly/birth defect (*n* = 1; 0.36%), and other medically important conditions (*n* = 37; 13.26%).

**Figure 2:**
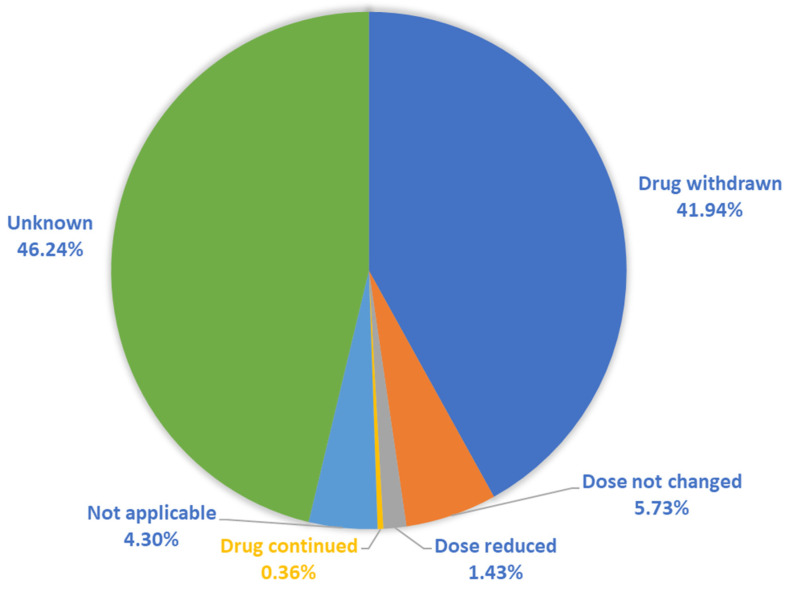
Actions taken for antimicrobial-related ADEs.

The actions taken when experiencing antimicrobial-related ADEs were to withdraw the drug (*n* = 117; 41.94%), not change the dose (*n* = 16; 5.73%), reduce the dose (*n* = 4; 1.43%), or continue with the drug (*n* = 1; 0.36%) (Figure 2).

### 2.5. Types of Antimicrobial-Related ADEs

Antimicrobial-related ADEs were categorized according to the MedDRA system organ classification (Table 4). The most commonly reported antimicrobial-related ADEs were those related to skin and subcutaneous tissue disorders (*n* = 105; 19.48%), followed by general disorders and administration conditions (*n* = 88; 16.33%), gastrointestinal disorders (*n* = 69; 12.8%), and nervous system disorders (*n* = 43; 7.98%). The least commonly reported antimicrobial-related ADEs were those related to psychiatric disorders (*n* = 3; 0.56%), ear and labyrinth disorders (*n* = 3; 0.56%), and reproductive system and breast disorders (*n* = 2; 0.37%).

Of the ADEs related to skin and subcutaneous tissue disorders, a rash (*n* = 58; 10.76%) was the most commonly reported, followed by urticaria (*n* = 22; 4.08%), pruritus (*n* = 14; 2.6%), and erythema (*n* = 11; 2.04%). Furthermore, in individuals who experienced ADEs related to general disorders and administration site conditions, the most frequently reported antimicrobial-related ADEs were edema (*n* = 28; 5.19%) and drug ineffectiveness (*n* = 28; 5.19%), followed by asthenia/fatigue/malaise (*n* = 11; 2.04%), and fever (*n* = 9; 1.67%).

Regarding gastrointestinal disorders, vomiting (*n* = 23; 4.27%) was the most reported ADE, followed by abdominal pain (*n* = 15; 2.78%), diarrhea (*n* = 12; 2.23%), and nausea (*n* = 7; 1.3%). In addition, seizures (*n* = 13; 2.41%) were the highest recorded ADE in patients who experienced nervous system symptoms. Among the patients who suffered from ADEs related to injury, poisoning, and procedure complications (*n* = 34; 6.31%), off-label use (*n* = 13; 2.41%) was the most reported.

It is worth mentioning that death was reported as an antimicrobial-related ADE in three ICSRs. However, the total number of patients who deceased was 13, as mentioned previously. Other antimicrobial-related ADEs regarding other system organ classes (SOCs) are reported in further detail (Table 4).

### 2.6. The Most Commonly Reported ADEs and Their Corresponding Antimicrobials

The most reported ADEs and their causative antimicrobial groups are presented (Table 5). Most of the patients who experienced rashes (*n* = 58) were following the consumption of third-generation cephalosporines (*n* = 11; 18.97%), tetracyclines (*n* = 7; 12.07%), fluoroquinolones (*n* = 6; 10.34%), and antimalarials (*n* = 5; 8.62%). Furthermore, most cases of renal and urinary-related ADEs (*n* = 30) were reported after the consumption of antivirals (*n* = 13; 43.33%), and glycopeptides (*n* = 11; 36.66%). In addition, more than half of the patients who experienced edemas (*n* = 28) were found to consume tetracyclines (*n* = 15; 53.57%). Drug ineffectiveness (*n* = 28) was reported the most in patients who consumed antivirals (*n* = 9; 32.14%) and tetracyclines (*n* = 8; 28.57%). Antivirals (*n* = 8; 29.63%) and tetracyclines (*n* = 6; 22.22%) were also responsible for most of the blood and lymphatic system-related ADEs (*n* = 27).

More than half of the patients who experienced vomiting (*n* = 23) were following the consumption of carbapenem (*n* = 7; 30.43%), macrolides (*n* = 4; 17.39%), and third-generation cephalosporins (*n* = 3; 13.04%). Moreover, almost all cases of urticaria (*n* = 22) were reported following tetracycline consumption (*n* = 17; 77.27%). Additionally, approximately half of the cases of allergy and hypersensitivity (*n* = 22) were after tetracycline (*n* = 8; 36.36%) and glycopeptide (*n* = 4; 18.18%) consumption. Finally, second-generation cephalosporin (*n* = 8; 38.1%) and antimalarial (*n* = 7; 33.33%) consumptions accounted for most cases of pregnancy, puerperium, and perinatal-related ADEs (*n* = 21).

### 2.7. The Most Consumed Antibiotic and Their Corresponding ADEs

The most consumed antibiotic (in order from left to right) in Jordan in 2020 [35] and their related ADEs are demonstrated (Table 6). Out of the total 539 reported antimicrobial-related ADEs, 125 (23.19%) were caused by the top ten consumed antibiotics. Out of the 125 ADEs, 28 were reported for the Access group antibiotics and 79 were for the Watch group antibiotics. Among the top ten consumed antibiotics, ciprofloxacin (Watch, *n* = 26) reported the highest number of ADEs, followed by cefuroxime (Watch, *n* = 24), and amoxicillin/clavulanic acid (Access, *n* = 22). It is worth mentioning that no antimicrobial-related ADEs were found to be associated with the consumption of doxycycline (Access) and clindamycin (Access).

In patients who consumed amoxicillin/clavulanic acid (Access), gastrointestinal-related ADEs (*n* = 11; 50%) were the most reported, with diarrhea (*n* = 4; 18.18%) accounting for most of the cases. Similarly, in patients who consumed clarithromycin (Watch), the most reported antimicrobial-related ADEs were gastrointestinal-related ADEs (*n* = 10; 71.43%), with abdominal pain (*n* = 3; 21.43%) and vomiting (*n* = 2; 14.23%) accounting for most of the cases.

The injury, poisoning, and procedure complications-related ADEs (*n* = 5; 29.41%) were mostly reported after azithromycin (Watch) consumption, and all cases were reported as off-label use (*n* = 5; 29.41%). Moreover, pregnancy, puerperium, and perinatal conditions (*n* = 8; 33.33%) and rashes (*n* = 4; 16.66%) were the most reported antimicrobial-related ADEs in patients who consumed cefuroxime (Watch). Among patients who consumed ciprofloxacin (Watch), skin and subcutaneous tissue ADEs (*n* = 8; 30.77%) and gastrointestinal ADEs (*n* = 7; 26.92%) were the most commonly reported.

## 3. Discussion

Prior research in Jordan had mostly examined the way in which healthcare professionals perceive and practice pharmacovigilance, including their knowledge, awareness, attitudes, and practices [21,36,37,38]. These investigations demonstrated that healthcare personnel had little understanding and awareness regarding the pharmacovigilance concepts [36,37]. Spontaneous ADR reporting by healthcare providers in Jordan was not common, despite participants in several studies revealing positive attitudes toward pharmacovigilance [21,38].

This retrospective study is the first to describe the trends in the frequency of ICSRs and the categories of ADEs linked to antimicrobial usage in Jordan. The reporting of ICSRs was significantly higher from 2019 onwards (a total of 219 cases), when compared to the period between 2003 and 2018 (a total of 60 cases). The highest reporting was noticed in 2022 (96 cases), followed by 2019 (65 cases), and 2021 (50 cases). This increase in the reporting of ADRs was influenced by the actions undertaken by the rational drug use and pharmacovigilance department (The National Pharmacovigilance Center) at the Jordan Food and Drug Administration (JFDA). These actions include updating the national pharmacovigilance guidelines in 2016, which encouraged ADR reporting and mandated healthcare practitioners to report ADRs. In 2021, a friendly-user electronic form was launched to ease the reporting process, alongside an awareness campaign directed towards the community and healthcare professionals, including videos and posters related to the concepts of pharmacovigilance. In addition, other factors played an important role in improving antimicrobial-related adverse reaction reporting in Jordan, including the implementation of the AMR-national action plan (2018–2022), updating the Essential Medicine List (EML) in 2021, which now determines how and when to use medication, and adapting the WHO AWaRe categorization, which is a useful tool for keeping track of antibiotic usage. Furthermore, continuous awareness campaigns and training programs were conducted by the JFDA to emphasize the importance of rational antimicrobial use, the risks of antimicrobial resistance, and the advantages of reporting any ADR that may be experienced by the patients. In 2022, the following has occurred: (i) the launch of a healthcare professional training program to strengthen the building capacity of pharmacovigilance and ADR reporting, and (ii) expanding regional pharmacovigilance centers to cover all the healthcare sectors in Jordan. Finally, there were many measures taken by JFDA to influence reporting of COVID-19 vaccine-related ADRs, including posters, press releases, and introducing electronic forms specific to COVID-19 vaccines in cooperation with the Uppsala Monitoring Centre, in both Arabic and English languages. The impact of these measures was more observable in 2021 and 2022 when the COVID-19 pandemic social restrictions were gradually reduced. Only 8 reports were recorded in 2020 (during the COVID-19 period). The decrease in reporting trends in 2020 is consistent with the previously described 5% reduction in the total consumption of antimicrobials in Jordan during 2020 compared to 2019 [35]. The marked reduction in some antibiotics may be explained by the fact that Jordan was under lockdown during the COVID-19 pandemic. This potentially caused a decrease in person-to-person transmission, possibly decreasing the incidence of respiratory tract infections, and fewer patient consultations, e.g., for self-limiting infections that would otherwise have resulted in an antibiotic prescription [35]. Another possible reason was that the healthcare system and workers were impacted heavily by the pandemic, which left them with less time and priority to report. A cross-sectional descriptive study conducted using the VigiFlow pharmacovigilance database in Sierra Leone between 2017 and 2021 found that the number of ICSRs was the highest in 2017 and 2019 (336 and 218 cases, respectively) [39]. Moreover, a decrease in reporting was noticed in 2020 (8 cases); however, this reduction continued into 2021 (3 cases), unlike in our study. The increase in reporting in 2017 and 2019 was explained by the mass medication programs during that period [39].

The majority of antimicrobial-related ADEs in our study were for tetracyclines (18.74%), fluoroquinolones (10.02%), third-generation cephalosporines (8.91%), and carbapenems (7.79%). On the other hand, the lowest adverse reactions were for antiprotozoals (0.19%) and other antibacterials (0.19%). In this study, the most common system organ classes (SOCs) that reported ADEs were skin and subcutaneous tissue disorders (19.48%), general disorders and administration site conditions (16.33%), gastrointestinal disorders (12.8%), and nervous system disorders (7.98%). In a study by Thomas et al., a total of 875 antimicrobial-related ADRs were identified [39]. Moreover, the most reported antimicrobial-related ADRs based on the SOCs were gastrointestinal disorders (*n* = 337, 38.51%), nervous system disorders (*n* = 167, 19.09%), general disorder and administration site conditions (*n* = 130, 14.86%), and skin and subcutaneous tissue disorders (*n* = 98, 11.2%). It is clearly noted that the top four SOCs are the same in our study as in the previously mentioned study, with only differences in the order.

The UMC issued a paper in 2017 in which it was agreed that AMR is a neglected adverse event of antimicrobial usage [40]. In this report, the larger disproportional reporting of antimicrobial treatment failure forced researchers to distinguish between two significant public health hazards: resistance and/or poor-quality medications. Thus, emphasizing the importance of using pharmacovigilance databases in detecting suspected AMR [28]. Based on that, Habarugira et al. designed a study to identify ADRs that suggest suspected antimicrobial resistance, ineffectiveness, and inappropriate use [41]. They extracted ADRs that are relevant to AMR-related events using a VigiAccess search between June and December 2018 and found that 5435 AMR-related ADRs were related to injury, poisoning, and procedure complications. The most frequently reported ADRs of the aforementioned category were related to off-label uses (*n* = 1455; 26.77%), unapproved indications (*n* = 1026; 18.88%), contraindicated product administration (*n* = 250; 4.6%), prescription errors (*n* = 196; 3.61%), and medication errors (*n* = 27; 0.5%). In our study, a total of 34 antimicrobial-related ADEs were related to injury, poisoning, and procedure complications. In the aforementioned category, the most commonly reported ADEs were related to off-label uses (*n* = 13; 38.24%), incorrect route of administration (*n* = 10; 29.41%), unapproved indications (*n* = 6; 17.65%), drug interactions (*n* = 2; 23.53%), and overdoses (*n* = 2; 23.53%).

In this study, we have assessed the antimicrobial agents responsible for the most commonly reported ADEs. Interestingly, tetracyclines were the most common agents reported with cases of edema (15 out of 28), urticaria (17 out of 22), and allergy and hypersensitivity (8 out of 22). Moreover, this type of antimicrobial was the second most common agent associated with rashes (7 out of 58), and drug ineffectiveness (8 out of 28). Such findings might be due to the common tetracycline adverse effects, and this point warrants further research assessments in the future. Antivirals were mostly reported to be associated with cases of renal and urinary disorders (13 out of 30) and blood and lymphatic system disorders (8 out of 27).

In a study conducted by Azzam et al., data regarding antimicrobial consumption in Jordan between 2019 and 2020 were collected from the JFDA using an Excel template provided by the WHO [35]. We used the results of this study regarding the most consumed antibiotics in Jordan to describe the relationship between antibiotic-related ADR reporting and the consumption of antibiotics, as well as the relationship between ADR reporting and the WHO AWaRe categories. We found that 125 antimicrobial-related ADEs were related to the top ten-consumed antibiotics in Jordan. Ciprofloxacin (Watch), which was the sixth most consumed antibiotic, reported the highest number of antibiotic-related ADEs. Moreover, cefuroxime (Watch), the fourth most consumed antibiotic, recorded the second-highest number of antimicrobial-related ADRs. Finally, amoxicillin/clavulanic acid (Access), the most commonly consumed antibiotic, was the third most common antibiotic with reported ADEs.

Four out of the top ten most consumed antibiotics were in the Access group (amoxicillin/clavulanic acid, amoxicillin, doxycycline, and clindamycin) and six were in the Watch group (azithromycin, cefuroxime, clarithromycin, ciprofloxacin, cefixime, and levofloxacin). The number of ADRs reported in the Watch group (*n* = 97; 77.6%) was higher than those reported in the Access group (*n* = 28; 22.4%). In one study, the percentage of ADRs reported for the Watch group (65.01%) was higher than those reported in the Access (33.19%) and Reserve (1.8%) groups, which is similar to our results [41].

This study is the first nationwide study to describe antimicrobial-related ADEs. In addition, we have evaluated the trends in reporting ADEs through an expansive time period (2003–2022). Furthermore, we described the relationship between the WHO AWaRe category of antibiotics and the reporting of ADEs, which provides a starting point for future assessments and helps create synergies between operational research and routine monitoring.

One of the study’s limitations was the lack and incompleteness of data regarding the duration between the onset and the reporting of antimicrobial-related ADEs. Some data regarding the age, gender, reporter qualification, seriousness of the ADEs, and actions undertaken relating to the antimicrobial-related ADE were missing. In addition, more work is needed on enhancing the quality of the received reports to allow more assessment of the received reports, i.e., Adverse Drug Reaction Probability Scale, and signal generation. Finally, and in relation to the deaths encountered by the patients, certain causality between the medicinal products and death could not be confirmed. However, the role of related medical products cannot be ruled out; most of the cases were not medically confirmed to be related to the consumed antimicrobials.

In conclusion, ADEs were higher in patients who received the Watch group antibiotics, highlighting the need to monitor and rationalize their use. The reporting of ADRs in Jordan increased after the implementation of various regulatory actions by the JFDA. However, the number of reported ADRs is still low and action plans should be implemented to encourage the reporting of antimicrobial-related ADRs, which can inform antimicrobial stewardship and improve pharmacovigilance systems in the country.

## 4. Materials and Methods

### 4.1. Study Design

This is a cross-sectional study of ICSRs submitted to the national pharmacovigilance center in Jordan, which was established in 2002. Reports of antimicrobial-related ADEs between 2003 and 2022 were included in this study.

### 4.2. Setting

Jordan Food and Drug Administration (JFDA) was founded in 2003 as an autonomous institution of the public sector and serves as the primary national responsible authority for assuring food safety and quality, as well as for medicines safety and efficacy [42]. Patients and healthcare providers in Jordan can report the occurrence of ADRs by using different available reporting methods, including electronic reporting using the JFDA webpage, calling JFDA, using the dedicated email address, or reporting back to the concerned company. In 2015, the first electronic report form was developed by JFDA. However, this trial was not successful since it was not well-developed, i.e., difficult to be used by the primary reporters and less accessible since it requires information to be entered using the JFDA website. In 2021, the Uppsala Monitoring Centre developed an electronic reporting tool that was easy to use and was translated into the Arabic language. There were multiple tools used for ADR reporting before the electronic form; the JFDA website, yellow form, the Council for International Organizations of Medical Sciences (CIOMS), by phone, email, and through focal points in the Pharmacovigilance Peripheral Centers located in some hospitals and educational universities.

### 4.3. Search Criteria

Using the Jordan pharmacovigilance database, data were extracted using the following codes: J01 Antibacterials for systemic use (ATC2), J02 Antimycotics for systemic use (ATC2), J05 Antivirals for systemic use (ATC2), D01B Antifungals for systemic use (ATC3), A07AA Antibiotics (ATC4), and P01BA Aminoquinolines (ATC4).

### 4.4. Data Extraction

All data were accessed using a web-based pharmacovigilance management system (Vigiflow; Uppsala Monitoring Centre product), via JFDA personnel. Extracted data included: age, gender, number of suspect/interacting drugs, reported medication, WHO drug trade name, WHO drug active ingredient variant, role, indication, dose, dose unit, dosage regimen, route of administration, start date, end date, action taken with drug batch number, term reported to UMC, mapped term, MedDRA preferred term, the seriousness of ADE, seriousness criteria of ADE, reporter qualification, and outcome.

### 4.5. Data Analysis

ADEs were presented by the system organ classes (SOCs) using the medical dictionary for regulatory activities (MedDRA) classification [43]. Frequencies and percentages were used to describe the categorical variables. If the ADE was recorded in patients who received two or more antimicrobials, it was counted as an ADE for each antimicrobial. The most consumed antibiotic and their AWaRe categories in Jordan were determined based on a previously published article [35].

## Figures and Tables

**Table 1:** Demographics and general characteristics of antimicrobial-related ADEs in individuals (*n* = 279).

	Number	Percentage (%)
**Age**		
0–17	68	24.37
18–64	121	43.37
≥ 65	24	8.6
NA	66	23.66
**Gender**		
Female	136	48.75
Male	100	35.84
NA	43	15.41
**Reporter qualification**		
Physician	103	36.92
Pharmacist	84	30.10
Other Health Professionals	36	12.90
Consumer/Non-Health Professional	22	7.89
Unknown	34	12.19

ADEs: adverse drug events, and NA: not applicable. Data are presented as whole numbers (percentages).

**Table 2:** Chemical subgroups of drugs and their ADRs included in the search.

Chemical Subgroup	Number of Products	Number of ADEs
Antifungals	12	35
Antivirals	8	24
Fluoroquinolones	5	54
Third-generation cephalosporines	5	48
Carbapenems	4	42
Macrolides	3	36
Other beta-lactam antibacterials	3	15
Tetracyclines	2	101
Second-generation cephalosporins	2	26
Aminoglycosides	2	3
Glycopeptide	1	28
First-generation cephalosporins	1	7
Penicillin with extended spectrum	1	6
Beta-lactamase resistant penicillin	1	3
Polymyxins	1	23
Antimalarials	1	24
Combinations of penicillin, incl. beta-lactamase inhibitors	1	24
Other cephalosporins and penems	1	15
Sulfonamides and trimethoprim	1	7
Aminoquinolines	1	3
Combinations of antibacterial	1	8
Other antibacterial	1	1
Antimycobacterial	1	5
Antiprotozoals	1	1
Total	60	539

ADEs: adverse drug events. Data are presented as whole numbers (percentages).

**Table 3:** Seriousness of antimicrobial-related ADEs.

	Number	Percentage (%)
**Seriousness of ADE**		
Yes	120	43.01
No	132	47.31
Unknown	27	9.68
**Seriousness criteria of ADE**		
Caused/prolonged hospitalization	58	20.79
Death	13	4.66
Life-threatening	10	3.58
Disabling/incapacitating	1	0.36
Congenital anomaly/birth defect	1	0.36
Other medically important conditions	37	13.26
Unknown	159	56.99

ADEs: adverse drug events. Data are presented as whole numbers (percentages).

**Table 4:** Types of antimicrobial-related ADEs in individuals (*n* = 279), according to the MedDRA system organ classification reported by the VigiBase in Jordan during 2003–2022.

Adverse Drug Events	Number of ADEs	Percentage of the Total ADEs (%)
**Total ADEs**	539	100
**Skin and subcutaneous tissue disorders**	105	19.48
Rash	58	10.76
Urticaria	22	4.08
Pruritus	14	2.60
Erythema	11	2.04
**General disorders and administration condition**	88	16.33
Facial edema/periorbital edema/peripheral edema	28	5.19
Drug ineffective	28	5.19
Asthenia/fatigue/malaise	11	2.04
Fever	9	1.67
Other (chills, sweating, hotness, flushing, pallor, jaundice, and lymphadenopathy)	12	2.23
**Gastrointestinal disorders**	69	12.8
Vomiting	23	4.27
Abdominal pain	15	2.78
Diarrhea	12	2.23
Nausea	7	1.30
Other (eructation, constipation, gastritis, hematemesis, taste disorders, tongue pigmentation, and pancreatitis)	12	2.23
**Nervous system disorder**	43	7.98
Seizure	13	2.41
Dizziness	8	1.48
Headache	6	1.11
Altered LOC	5	0.93
Other (stroke, gait disturbance, memory impairment, paresthesia, photophobia, somnolence, and others)	11	2.04
**Injury, poisoning, and procedure complications**	34	6.31
Off-label use	13	2.41
Incorrect route of administration	10	1.86
Unapproved indication	6	1.11
Overdose	2	0.37
Drug interaction	2	0.37
Treatment non-compliance	1	0.19
**Renal and urinary disorders (AKI, abnormal renal function, abnormal electrolytes, glycosuria, and others)**	30	5.57
**Cardiac disorders**	28	5.19
Hypotension	10	1.86
Palpitations	4	0.74
Chest pain or discomfort	4	0.74
Myocardial infarction	3	0.56
Tachycardia	3	0.56
Other (syncope, AV block, cardiac arrest, syncope)	4	0.74
**Blood and lymphatic system disorders (anemia, leukocytosis, leukopenia, thrombocytosis, thrombocytopenia, bleeding, and coagulation)**	27	5.01
**Immune system disorders (allergy and hypersensitivity)**	22	4.08
**Respiratory, thoracic, and mediastinal disorders**	21	3.90
Dyspnea	14	2.60
Other (cough, cyanosis, tachypnea, and apnea)	7	1.30
**Pregnancy, puerperium, and perinatal conditions (fetal exposure, premature labor, and others)**	21	3.90
**Musculoskeletal and connective tissue disorders (arthralgia, myalgia, and stiffness)**	9	1.67
**Product issues**	9	1.67
**Hepatobiliary disorders (AST, ALT, and bilirubin)**	9	1.67
**Metabolism and nutrition disorders (hypoglycemia)**	7	1.30
**Eye disorders (blindness, visual impairment, corneal edema, keratitis, and scleromalacia)**	6	1.11
**Psychiatric disorders (narcolepsy and suicide attempt)**	3	0.56
**Ear and labyrinth disorders (vertigo, hearing impairment, and others)**	3	0.56
**Death** *	3	0.56
**Reproductive system and breast disorders (mastoiditis)**	2	0.37

* Death was reported as an adverse drug event in 3 cases, although in the seriousness criteria section, another 13 cases suffering from other adverse events were eventually deceased. ADEs: adverse drug events. Data are presented as whole numbers (percentages).

**Table 5:** The most common antimicrobial-related ADEs and their corresponding antimicrobial.

Antibiotic Group	Rash (*n* = 58)	Renal and Urinary Disorders (*n* = 30)	Facial Edema/Periorbital Edema/Peripheral Edema (*n* = 28)	Drug Ineffective (*n* = 28)	Blood and lymphatic System Disorders (*n* = 27)	Vomiting (*n* = 23)	Urticaria (*n* = 22)	Immune System Disorders (Allergy and Hypersensitivity) (*n* = 22)	Pregnancy, Puerperium, and Perinatal Conditions (*n* = 21)
Aminoglycosides	-	-	-	3	-	-	-	-	-
Aminoquinolines	-	-		-	-	-	-	1	1
Antifungals	2	1	1	3	3	-	-	-	-
Antimalarials	5	-	-	-	-	-	-	-	7
Antimycobacterial	2	-	-	-	1	-	1	-	-
Antiprotozoals	1	-	-	-	-	-	-	-	-
Antivirals	-	13	-	1	8	-	-	-	-
Beta-lactamase Resistant penicillin	-	-	-	-	-	1	-	-	-
Carbapenems	1	-	-	9	-	7	-	1	1
Tetracyclines	7	-	15	8	6	1	17	8	-
Fluoroquinolones	6	-	5	-	3	-	2	1	-
Penicillin with extended spectrum	2	-	-	-	-	1	-	-	-
Combinations of penicillin, incl. beta-lactamase inhibitors	1	-	-	-	-	2	-	-	-
First-generation cephalosporins	3	-	-	-	-	1	-	-	-
Second-generation cephalosporins	4	-	1	-	2	1	-	2	8
Third-generation cephalosporines	11	-	3	3	-	3	2	2	4
Other cephalosporins and penems	-	-	-	-	2	1	-	-	-
Glycopeptide	4	11	1	1	1	1	-	4	-
Macrolides	-	-	1	-	-	4	-	1	-
Other beta-lactam antibacterials	4	1	-	-	-		-	1	-
Polymyxins	2	3	-	-	1	-	-	1	-
Sulfonamides and trimethoprim	2	1	-	-	-	-	-	-	-
Combinations of antibacterial	1	-	1	-	-	-	-	-	-

ADEs: adverse drug events. Data are presented as whole numbers (percentages).

**Table 6:** Antimicrobial-related ADEs of the most consumed antibiotics in 2020.

	Amoxicillin/Clavulanic Acid	Amoxicillin	Azithromycin	Cefuroxime	Clarithromycin	Ciprofloxacin	Cefixime	Doxycycline	Clindamycin	Levofloxacin
**AWaRe Classification**	**ACCESS**	**ACCESS**	**WATCH**	**WATCH**	**WATCH**	**WATCH**	**WATCH**	**ACCESS**	**ACCESS**	**WATCH**
**Gastrointestinal disorders**	11	2	4	3	10	7	-	**No Adverse Reactions**	**No Adverse Reactions**	-
Abdominal pain	2	-	1	-	3	4	-			-
Abnormal taste	-	-	-	-	1	-	-			-
Belching	-	-	-	1	-	2	-			-
Constipation	1	-	-	-	-	-	-			-
Diarrhea	4	1	1	1	1	1	-			-
Gastritis	-	-	-	-	1	-	-			-
Hematemesis	2	-	-	-	-	-	-			-
Nausea	1	-	1	-	1	-	-			-
Pancreatitis	-	-	-	-	1	-	-			-
Vomiting	1	1	1	1	2	-	-			-
**General disorders**	2	-	1	1	-	2	-			3
Facial edema/periorbital edema/peripheral edema	-	-	1	1	-	1	-			1
Fever	2	-	-	-	-	-	-			-
Hemorrhage	-	-	-	-	-	-	-			2
Hot flush	-	-	-	-	-	1	-			-
Lymphadenopathy	-	-	-	-	-	-	-			-
**Injury, poisoning, and procedure complications**	2	1	5	-	-	-	2			3
Incorrect route of administration	1	-	-	-	-	-	1			1
Off-label use	-	1	5	-	-	-	1			
Overdose	1	-	-	-	-	-				
Drug interaction	-	-	-	-	-	-	-			2
**Nervous system disorder**	3	-	-	1	2	4	-			-
Dizziness	1	-	-	-	2	1	-			-
Headache	1	-	-	-	-		-			-
Loss of consciousness	1	-	-	1	-	3	-			-
**Respiratory, thoracic, and mediastinal disorders**	1	-	1	2	-	-	-			-
Apnea	-	-		1	-	-	-			-
Arthralgia	1	-			-	-	-			-
**Cardiac disorders**	-	-	4	2	1	-	-			-
Chest pain	-	-	2		1	-	-			-
Dyspnea	-	-	1	1	-	-	-			-
Hypotension	-	-	1	2	-	-	-			-
Palpitation	-	-	1		-	-	-			-
**Skin and subcutaneous tissue disorders**	1	2	-	4	-	8	2			1
Erythema	-	-	-	-	-	1	-			-
Pruritus	-	-	-	-	-	3	-			-
Rash	1	2	-	4	-	2	2			1
Urticaria	-	-	-	-	-	2	-			-
Lymphadenopathy	-	-	-	-	-	-	-			-
**Ear and labyrinth disorders (hypoacusis)**	-	-	-	-	1	-	-			-
**Hepatobiliary disorders (abnormal INR)**	-	-	-	-	-	-	-			1
**Immune system disorders (hypersensitivity)**	-	-	1	2	-	-	-			1
**Metabolism and nutrition disorders (hypoglycemia)**	-	-	-		-	5	-			-
**Product issues**	-	-	1		-	-	-			-
**Pregnancy, puerperium, and perinatal conditions**	-	-	-	8	-	-	3			-
**Psychiatric disorders (suicide attempt)**	1	-	-	1	-	-	-			-
**Reproductive system and breast disorders (mastoiditis)**	1	1			-	-	-			-
**Total ADEs**	22	6	17	24	14	26	7	0	0	9

ADEs: adverse drug events. AWaRe classification: WHO Access, Watch, and Reserve classifications of antibiotics for evaluation and monitoring of use. Data are presented as whole numbers (percentages).

## Data Availability

Data are available on reasonable request and in line with permission approval processes from the JFDA.
